# Chiropractic international research collaborative (CIRCuit): the development of a new practice-based research network, including the demographics, practice, and clinical management characteristics of clinician participants

**DOI:** 10.1186/s12998-025-00568-1

**Published:** 2025-01-10

**Authors:** Kenneth J Young, Sasha Aspinall, Silvano Mior, Jordan Gliedt, Joseph Spencer, Christoffer Børsheim, Jennifer Nash, Melinda Ricci, Jonathan Shurr, Iben Axén

**Affiliations:** 1 University of Central Lancashire, Preston, UK; 2 University of Johannesburg, Doornfontein, South Africa; 3 School of Allied Health College of Health and Education, Murdoch University, Perth, Australia; 4 Canadian Memorial Chiropractic College, Toronto, Canada; 5 Institute for Disability and Rehabilitation Research, University of Toronto, Toronto, Canada; 6 Institute of Health Policy, Management and Evaluation, University of Toronto, Toronto, Canada; 7 Faculty of Health Sciences, Ontario Tech University, Ontario, Canada; 8 Department of Neurosurgery, Medical College of Wisconsin, Milwaukee, WI USA; 9 Research Facilitation and Delivery Unit Applied Health Research Hub, University of Central Lancashire, Preston, UK; 10 Private practice, Bergen, Norway; 11 Department of Anesthesia, McMaster University, Hamilton, ON Canada; 12 Success Chiropractic Practice Serpentine Jarrahdale Chiropractic Practice, Whitby, WA Australia; 13 Private practice, Lancashire, UK; 14 Karolinska Institutet Institute of Environmental Medicine, Nobels v. 13, 177 77, Stockholm, Sweden; 15 The Norwegian Chiropractors’ Research Foundation «Et liv i bevegelse», Lilleakerveien 31, Oslo, 0283 Norway

**Keywords:** Practice-based research network, Chiropractic, Survey

## Abstract

**Objectives:**

To describe the structure and development of a new international, chiropractic, practice-based research network (PBRN), the Chiropractic International Research Collaborative (CIRCuit), as well as the demographic, practice, and clinical management characteristics of its clinician participants. An electronic survey was used to collect information on their demographics, practice, and clinical management characteristics from clinicians from 17 October through 28 November 2022. Descriptive statistics were used to report the results.

**Background:**

PBRNs are an increasingly popular way of facilitating clinic-based studies. They provide the opportunity to collaboratively develop research projects involving researchers, clinicians, patients and support groups. We are unaware of any international PBRNs, or any that have a steering group comprised of equal numbers of clinicians representing the different international regions.

**Results:**

77 chiropractors responded to the survey (0.7% of EBCN-FB members). 48 were men (62%), 29 women (38%). Thirty-six (47%) were in North America, 18 (23%) in Europe, and 15 (19%) in Oceania. Participants reported predominantly treating musculoskeletal issues, often with high-velocity, low-amplitude spinal manipulation (95%), but also with soft tissue therapy (95%), exercise (95%), and other home care (up to 100%).

**Methods:**

The development of CIRCuit is described narratively. Members of the Evidence-Based Chiropractic Network Facebook group (EBCN-FB) were invited to become clinician participants by participating in the survey.

**Conclusions:**

This paper describes the development of a new PBRN for chiropractors. It offers a unique opportunity to facilitate the engagement of clinical chiropractors with research, as well as for academics to readily be able to access an international cohort of clinicians to collaboratively develop and conduct research. Although the results of the survey are not statistically generalisable, the initial cohort of CIRCuit clinician participants use similar techniques on similar types of conditions as the profession at large. The international structure is unique among PBRNs and offers the opportunity to help develop innovative research projects.

**Supplementary Information:**

The online version contains supplementary material available at 10.1186/s12998-025-00568-1.

## Background

Conducting high-quality research is necessary to ensure optimal patient care (1). Practice-based research is becoming increasingly popular because it offers an approach for undertaking studies potentially more relevant to frontline clinical practice [2]. One way of strategically organising this approach to research is within a practice-based research network (PBRN) framework. A PBRN is a collective group of healthcare providers and researchers, united by a vision and goal to participate in scientific investigations and collect data from within the providers’ own clinics [3, 4]. PBRNs typically participate in studies which aim to answer community-based research questions [4]. The clinical setting for PBRN research differs from the traditional university laboratory, in which investigators undertake studies using a limited pool of participants, in an artificial clinical environment. Thus, PBRNs provide a structure that facilitates the collection of clinical data to answer relevant research questions and thereby may improve the generalisability of results.

PBRNs are part of professional partnerships between researchers, clinicians, and support personnel including administrative staff and volunteers. These partnerships are maintained by defined, specific processes, grounded by a variety of infrastructures [3]. PBRNs can potentially enhance participant recruitment, increase stakeholder engagement (e.g., clinicians and patients), and even help produce changes to systems beyond research or academia [5] For example, ‘in Project TEAL: Tribal Efforts Against Lead, their partnership activities inspired some members to work on related projects addressing lead poisoning’ [6]. Chiropractic PBRNs have helped augment the research environment in the profession. For example, the CRUNCh PBRN in the UK provided a springboard for other projects e.g. facilitating nested PhD projects (https://crc-uk.org/when-it-comes-to-the-crunch/). Other PBRNs explored attributes of practitioner and patient populations. For example, the ACORN PBRN in Australia helped in a study on chiropractors’ use of nutritional guidance [7] and the International Chiropractic Pediatric Association (ICPA) PBRN has investigated presenting complaints for paediatric patients reporting to chiropractic practices [8].

Furthermore, when the results of studies are reported, dissemination of new information into practice can be facilitated through the PBRN, thereby potentially decreasing the time from concept to implementation [9–11]. A PBRN may also be seen as building cohesion among clinicians and researchers where there is a mutual appreciation of each other’s worlds (1).

PBRNs may take a variety of forms. Some PBRNs undertake research directly, some fund research undertaken by research groups looking to collect clinic-based data, others maintain databases for projects, or they may use a combination of these [12].

The chiropractic profession has recognised the potential advantages of PBRNs. There are examples of chiropractic PBRNs that have contributed to research and aided in its dissemination [5, 13–15]. However, developing and more importantly sustaining, a chiropractic PBRN is a challenging prospect [3]. One major issue is the recruitment and engagement of busy clinicians; other challenges include fundraising, meeting administrative needs, building and maintaining relationships with researchers and clinicians, and achieving maturation to the stage of facilitating large studies [9, 16, 17].

Given the increasing global burden of musculoskeletal conditions [18–20], an international PBRN may provide data extending beyond specific country borders, contributing unique international evidence. The aims of this paper are to:

1. Describe the development and features of a new international PBRN, and.

2. Describe the demographics, practice, and clinical management characteristics of clinicians who volunteered to join the new PBRN.

## Methods

### Developing a PBRN structure

The idea of developing a new international chiropractic PBRN grew from a collaboration between a diverse group of stakeholders, including chiropractic clinicians, academics, and leaders of the Evidence-Based Chiropractic Network (EBCN - https://www.facebook.com/groups/evidencebasedchiropractors). The overarching purpose was to facilitate the realisation and dissemination of chiropractic focused community-engaged research across the world.

The idea for a new PBRN started as a discussion between colleagues at universities and the EBCN. A steering committee was formed from each of the following regions in the world: Europe, Oceania, and North America. Countries represented included the United Kingdom (UK), Sweden, Norway, United States of America (USA), Canada, and Australia. There was one clinician and one researcher each, from UK, the European Union, USA, Canada, and Australia. The researchers and clinicians are a mix of mid-career to later career.

The steering committee identified five key principles for this international chiropractic PBRN. The first principle was that the steering committee would be comprised of equal numbers of clinicians and researchers. The second was internationality (a form of multi-centredness), which was seen as key to attracting research projects that included diverse groups of participants and could explore regional differences in chiropractic practice. Internationality also improves the chance of creating datasets of sufficient sizes to be able to draw valid conclusions. The third principle was independence. It was seen as critical that no one association or organisation should influence PBRN decisions. The fourth was that research supported by the PBRN should be carried out in the public interest, i.e., public health priorities would guide decisions regarding which projects to support. The fifth was simplicity. Researchers will be invited to submit project proposals, which will be checked by the CIRCuit scientific review committee for rigour, relevance to public health priorities, and achievability. Currently, there are five members of the scientific review committee, 2 women and 3 men, ranging from 8 to 30 years of experience, all in urban or suburban areas, and all have PhDs. Collectively, they have expertise in basic science, clinical, and educational research using both qualitative and quantitative methods. Scientific review committee members’ may also call upon their own professional networks to invite external reviewers to assist with evaluating research proposals as necessary.

If a project is approved, invitations will be sent on behalf of the researchers to clinicians with relevant practice characteristics. Clinicians are under no obligation to participate in any particular research project, but then may contact the researchers directly to participate in the project. CIRCuit will not conduct or fund research itself. These five principles were thought to improve the likelihood for stability and sustainability of the PBRN.

The steering committee voted on and adopted a name for the PBRN: Chiropractic International Research Collaborative, or CIRCuit for short. Plans were developed to disseminate results of studies by providing links to research developed through CIRCuit on the CIRCuit web site, as appropriate and in conformity with intellectual property rights. Further group discussions were held to explore the need for and mechanism of applying for charity status as well as a fundraising strategy.

### Recruiting and surveying clinician participants

#### Preparation for the survey

The next step in developing the PBRN was to recruit clinician participants, chiropractors in whose clinics research projects would be undertaken. Recruitment would result in a database with clinician characteristics for future use. Given matters of privacy and confidentiality of collected data and its analysis, and with input from legal counsel, a data sharing agreement was drawn up by the University of Central Lancashire (UCLan) legal department between UCLan and CIRCuit so that the data collected by CIRCuit could be analysed and stored at UCLan. Informed consent was obtained from participants electronically at the beginning of the survey. Ethical approval for the study was obtained through the UCLan Health Research Ethics committee (Health0317). The participant information sheet included details about the risks and benefits of responding to the survey.

#### Participant population

The CIRCuit steering group decided that to best carry out research in the public interest, clinician participants should demonstrate a commitment to evidence-based practice. Therefore, we used purposive sampling to invite participants from the population of membership in the EBCN. Participation in the survey was voluntary. Participants were notified that there was no further obligation to participate in any research project, but that only EBCN members who wished to undertake research should respond to the survey.

#### Instrument development

The steering committee of CIRCuit developed a questionnaire through an iterative process, resolving conflicts through discussion. The aim was to obtain descriptive data on participants (age, gender, academic degrees, specialist knowledge, professional activities, country and language) and the characteristics of their practices (years in practice, location of practice, number of hours in practice, number of patients, types of associates, access to imaging, type of patient records, type of payment). In addition, participants were asked to describe their patient populations (reasons for seeking care and caring for special groups) and the therapeutic interventions that they used (chiropractic techniques and manual therapies, other therapeutic methods, education of patients, and referral patterns). The full questionnaire can be found in Additional File 1.

#### Participant recruitment and data collection

We used a herald notice and weekly follow-up invitations to recruit participants online [21]. A link to the questionnaire, hosted by JISC Surveys (Joint Information Systems Committee – Bristol, UK) was distributed through the EBCN Facebook group on 17 October 2022. The link to the questionnaire remained open for 6 weeks to maximise responses. Posts to the EBCN Facebook group, on the CIRCuit Facebook timeline, and on the Facebook timelines of individual CIRCuit members were used to promote the study. In addition, personal reminders were sent by CIRCuit steering group members to eligible participants in their networks. The survey was also advertised on the CIRCuit Facebook page and the CIRCuit website.

#### Sample size

Based on previous studies of chiropractors’ participation in research [22, 23], we estimated that 1% of the 11,700 EBCN members would respond to the survey.

#### Data analysis

The results were exported from the JISC platform into Microsoft Excel (Microsoft, Redmond, CA) and SPSS Statistics 29 (Statistical Product and Service Solutions – IBM, Armonk, NY) for analysis. Descriptive statistics were used to summarise the data. We compared the age, gender identity, and country of practice between respondents to our survey and members of the EBCN Facebook group, from which the sample was drawn. Data for the EBCN group was obtained from Facebook on 22 December 2022.

Because of the low response rate, we did not perform inferential statistics, therefore we have not reported correlations between, e.g., age and research involvement or country and highest degree attained.

#### Reporting of results

Results were reported narratively, using tables and figures to supplement and visualise items.

## Results

### Results of CIRCuit structural development

CIRCuit developed a website (www.circuitpbrn.org*)* and a Facebook page and was registered as a Charitable Incorporated Organisation (CIO) in the UK (#1195528). A Patreon account to facilitate and track donations was also created. Finally, CIRCuit also identified a flagship project that will see the first use of its system.

In line with principles of simplicity and sustainability, the structure of CIRCuit was developed to function as follows: Researchers who would like to conduct a study using practice-based data collection contact CIRCuit. The CIRCuit scientific review committee evaluate the project in the context of the CIRCuit mission and charitable duty to facilitate research that serves a public health or public interest function. The database of clinician participants is then searched for those practitioners with suitable demographics and practice characteristics to undertake that particular project. The appropriate clinician participants are invited to respond to the research call. Those amenable and available for the timeframe of the study are then be put in contact with the researchers for data collection and if appropriate, collaborative project development. CIRCuit will not participate directly in conducting or funding research projects.

### Results of clinician affiliate survey

There were 77 responses to the survey (0.7% of 11,700 EBCN members).

#### Practitioner characteristics

Detailed practitioner characteristics are presented in Table 1. The survey respondents practiced predominantly in North America, Europe, and Oceania. They had been in practice for a mean of 14.6 years (SD 10.5), usually held one qualification only – (95%), and the majority routinely consulted patients in the English language (86%).

**Table 1:** Practitioner characteristics

PRACTITIONER CHARACTERISTICS		RESPONSE (*n* = 77)
Age in years, mean (SD)		43 (SD 11.5)
Gender identity, n (%)	Woman	29 (38%)
	Man	48 (62%)
	Non-binary or other	0 (0%)
Region of practice, n (%)	Africa	1 (1%)
	Asia	5 (6%)
	Europe	18 (23%)
	North America	36 (47%)
	Oceania	15 (19%)
	South America	2 (3%)
Years of practice, mean (SD); median (IQR)		14.6 (SD 10.5); 12 (IQR 14)
Years of practice, n (%)	0–4	13 (17%)
	5–9	17 (22%)
	10–19	24 (31%)
	20–29	12 (16%)
	30–39	10 (13%)
	40+	1 (1%)
Health professions, n (%)*	Chiropractor	77 (100%)
	Acupuncturist	1 (1%)
	Physical therapist	1 (1%)
	Emergency medical technician	1 (1%)
	Dietician/ nutritionist	1 (1%)
	Naturopath	1 (1%)
Languages routinely used for patient consultation, n (%)*	English	66 (86%)
	French	57
	Dutch	4 (5%)
	Norwegian	3(4%)
	Spanish	3(4%)
	Other	12 (16%)
Highest academic degree, n (%)	Bachelor	5 (6%)
	Masters	15 (19%)
	Doctor of Chiropractic	48 (62%)
	Doctor of Philosophy	9 (12%)
Other professional activities, n (%)*	Teaching	17 (22%)
	Research	28 (36%)
	Paid or volunteer work for chiropractic organisation	30 (39%)
	Volunteer chiropractic practice	15 (19%)

*Sub-groups may sum to more than 77 (100%) as participants were able to enter multiple options

For comparison, the EBCN group at large, from which participants were recruited, had a mean age of 38 years, with 42% women and 58% men. A total of 39% were from the USA, 26% from Canada, 10% from Australia, 0.7% from the UK, and the remainder from other countries. Hence the CIRCuit survey respondents had a slightly older mean age, a lower proportion of women, a lower proportion of practitioners from the USA, and a higher proportion from Canada, Australia, and the UK than the EBCN group overall.

#### Practice characteristics

Detailed practice characteristics are presented in Table 2. Over the prior three months, the average direct patient contact hours per week was 34.5 h (SD 47.3), the average patient visits per week was 55.5 (SD 33.0), and the average new patient visits per week was 4.9 (SD 3.4).

The majority of respondents reported practicing in urban settings (81%) and reported being in multi-disciplinary practices (48%), but large pluralities were in solo practices (38%), or multi-chiropractor practices (35%). Respondents who indicated that they worked in a multi-disciplinary practice were asked to specify which other types of practitioners also worked in the practice. Among those 37 respondents, the responses were massage therapist (22, 59%), physical therapist/physiotherapist (14, 38%), counsellor/psychologist (14, 38%), medical practitioner (12, 32%), dietician/nutritionist (11, 30%), fitness professional (10, 27%), and podiatrist (6, 16%). Ten respondents (27%) also selected the ‘other’ option and subsequent free text responses included acupuncturists (5, 13%) and traditional Chinese medicine practitioners (3, 8%). All responses to ‘other’ boxes can be found in Additional file 2.

The majority reported having no imaging facilities on-site (84%) and using primarily electronic recordkeeping (79%). Nearly all reported accepting payment in the form of private/patient pay (97%), with 27% exclusively accepting this type of payment, and the remainder accepting various other forms of payment.

**Table 2:** Practice characteristics

PRACTICE CHARACTERISTICS		RESPONSE (*n* = 77)
Average direct patient contact hours per week, mean (SD); median (IQR)		34.5 (SD 47.3); 28 (IQR 15)
Average direct patient contact hours per week, n (%)	0–9	3 (4%)
	10–19	12 (16%)
	20–29	26 (34%)
	30–39	22 (29%)
	40–49	12 (16%)
	50+	2 (3%)
Average total patient visits per week, mean (SD); median (IQR)		55.5 (SD 33.0); 50 (IQR 30)
Average total patient visits per week, n (%)	0–19	8 (10%)
	20–39	10 (13%)
	40–59	32 (42%)
	60–79	12 (16%)
	80–99	5 (6%)
	100+	10 (13%)
Average new patient visits per week, mean (SD); median (IQR)		4.9 (SD 3.4); 4 (IQR 3)
Average new patient visits per week, n (%)	0–4	39 (51%)
	5–9	29 (38%)
	10–14	6 (8%)
	15–19	3 (4%)
Geographic practice setting, n (%)*	Urban	63 (82%)
	Rural	24 (31%)
	Remote	2 (3%)
Types of practice, n (%)*	Solo practice	29 (38%)
	Muli-chiropractor practice	27 (35%)
	Multi-disciplinary practice	37 (48%)
	Hospital-based practice	4 (5%)
	Other	3 (4%)
Imaging facilities on-site, n (%)*	None	65 (84%)
	X-rays	6 (8%)
	Diagnostic ultrasound	6 (8%)
	MRI	2 (3%)
	CT	1 (1%)
Types of recordkeeping, n (%)*	Primarily electronic	61 (79%)
	Primarily paper-based	10 (13%)
	Combination of electronic and paper-based	7 (9%)
Types of payment routinely accepted, n (%)*	Private/patient pay	75 (97%)
	Private health insurance reimbursement	55 (71%)
	Public health insurance reimbursement	19 (25%)
	Worker’s compensation	21 (27%)
	Personal injury claims	23 (30%)

*Sub-groups may sum to more than 77 (100%) as participants were able to select multiple options

#### Patient management characteristics

Table 3 details the respondents’ reported frequencies for managing certain conditions, managing special populations, and referring patients for diagnostic imaging. Respondents commonly managed low back pain (99% responded ‘often’), neck pain (95% often), and mid back pain (87% often), and the majority reported never/rarely managing non-musculoskeletal disorders (74%). In terms of special populations, those commonly managed were older adults (81% often), athletes (51% often), and adolescents (35% often). Regarding the frequency of obtaining or referring for diagnostic imaging, x-ray was most common and computed tomography least common.

**Table 3:** Frequency of conditions managed, special populations managed, and Diagnostic Imaging Use

CHARACTERISTIC	FREQUENCY, *n* (%)			
	Often	Sometimes	Never/Rarely	Not applicable
Conditions managed				
Low back pain	76 (99%)	1 (1%)	0 (0%)	-
Mid back pain	67 (87%)	10 (13%)	0 (0%)	-
Neck pain	73 (95%)	4 (5%)	0 (0%)	-
Radicular symptoms	47 (61%)	30 (39%)	0 (0%)	-
Headaches	51 (66%)	21 (27%)	5 (6%)	-
Shoulder pain	49 (64%)	27 (35%)	1 (1%)	-
Elbow pain	14 (18%)	53 (69%)	10 (13%)	-
Wrist or hand pain	13 (17%)	49 (64%)	15 (19%)	-
Hip pain	50 (65%)	26 (34%)	1 (1%)	-
Knee pain	32 (42%)	43 (56%)	2 (3%)	-
Calf, ankle, or foot pain	17 (22%)	51 (66%)	9 (12%)	-
Sports injuries	35 (45%)	33 (43%)	9 (12%)	-
Postural disorders	32 (42%)	29 (38%)	16 (21%)	-
Non-musculoskeletal disorders	5 (6%)	15 (19%)	57 (74%)	-
**Special populations managed**				
Infants (≤ 1 year)	4 (5%)	14 (18%)	57 (74%)	2 (3%)
Children (2–11 yrs)	6 (8%)	41 (53%)	29 (38%)	1 (1%)
Adolescents (12–18 yrs)	27 (35%)	47 (61%)	3 (4%)	0 (0%)
Older adults (≥ 60 yrs)	62 (81%)	11 (14%)	1 (1%)	3 (4%)
Pregnant females	12 (16%)	49 (64%)	15 (19%)	1 (1%)
Athletes	39 (51%)	27 (35%)	9 (12%)	2 (3%)
Native/indigenous people	7 (9%)	26 (34%)	38 (49%)	6 (8%)
Disabled people	3 (4%)	37 (48%)	36 (47%)	1 (1%)
**Frequency of obtaining or referring for diagnostic imaging**				
X-ray	6 (8%)	55 (71%)	16 (21%)	-
Magnetic resonance imaging	2 (3%)	47 (61%)	28 (36%)	-
Diagnostic ultrasound	4 (5%)	35 (45%)	38 (49%)	-
Computed tomography	0 (0%)	23 (30%)	54 (70%)	-

Table 4 details other patient management characteristics including types of interventions used and practitioner referrals.

Respondents frequently reported they had expertise in the management of chronic pain (69%), headaches (66%), and athletic injuries (58%).

In terms of the types of interventions used, respondents overwhelmingly reported using high-velocity low-amplitude manipulation (95%) and soft-tissue therapies (95%), as well as a range of other manual therapies. The majority reported they did not use a specific chiropractic technique system (65%), though the most common technique systems were Activator Methods^®^ (22%) and Cox^®^ Flexion-Distraction (21%). Adjunct therapies included at-home exercise (95%), supervised exercise (58%), heat or cold therapy (45%), and dry needling or acupuncture (45%). In terms of topics the respondents routinely reported educating patients about, the most common were physical activity (100%), stress management (79%), and workplace modifications (79%).

Finally, respondents reported routinely referring patients to general practitioners (82%), massage therapists (64%), and physical therapists/physiotherapists (58%).

**Table 4:** Clinical care characteristics

MANAGEMENT CHARACTERISTICS		RESPONSE (*n* = 77)
Self-reported management expertise, n (%)*	Chronic pain	53 (69%)
	Headaches	51 (66%)
	Athletic injuries	45 (58%)
	Dizziness and vertigo	25 (32%)
	Pregnancy and post-partum pain	21 (27%)
	Paediatrics	4 (5%)
	Other	7 (9%)
	None	5 (6%)
Types of manual therapy routinely used, n (%)*	High-velocity low-amplitude manipulation	73 (95%)
	Instrument-assisted joint manipulation	42 (55%)
	Joint mobilisation	65 (84%)
	Flexion-distraction	31 (40%)
	Drop-piece	47 (61%)
	Pelvic blocking	24 (31%)
	Soft tissue therapy, trigger point therapy, or massage	73 (95%)
	Instrument-assisted soft tissue mobilisation	44 (57%)
	Other	9 (12%)
Chiropractic technique systems routinely used, n (%)*	Activator methods	17 (22%)
	Advanced Biostructural Correction Technique	1 (1%)
	Applied Kinesiology	3 (4%)
	Chiropractic Biophysics	2 (3%)
	Cox Flexion-Distraction	16 (21%)
	Gonstead Technique	10 (13%)
	Sacro-Occipital Technique	8 (10%)
	Thompson Technique	13 (17%)
	Webster Technique	5 (6%)
	Other	7 (9%)
	Do not use a technique system	50 (65%)
Adjunct therapies routinely used, n (%)*	At-home exercise	73 (95%)
	Supervised exercise	45 (58%)
	Heat or cold therapy	35 (45%)
	Rigid taping	13 (17%)
	Biomechanical taping	33 (43%)
	Dry needling or acupuncture	35 (45%)
	Orthotics	18 (23%)
	TENS	13 (17%)
	Laser therapy	15 (19%)
	Therapeutic ultrasound	11 (14%)
	Other	12 (16%)
	Do not use adjunct therapies	0 (0%)
Topics of routine patient education, n (%)*	Physical activity	77 (100%)
	Sleep hygiene	56 (73%)
	Stress management	61 (79%)
	Smoking, drugs, or alcohol	37 (48%)
	Weight management	33 (43%)
	Diet or nutrition	43 (56%)
	Pain science or pain education	64 (83%)
	Workplace modifications	61 (79%)
	Other	4 (5%)
	Do not use patient education	0 (0%)
Practitioners routinely referred to, n (%)*	Other chiropractor	22 (29%)
	Physical therapist/physiotherapist	45 (58%)
	Osteopath	5 (6%)
	General practitioner	63 (82%)
	Medical specialist	41 (53%)
	Dietician or nutritionist	19 (25%)
	Podiatrist	16 (21%)
	Occupational therapist	6 (8%)
	Speech pathologist	1 (1%)
	Counsellor or psychologist	30 (39%)
	Fitness professional	31 (40%)
	Massage therapist	49 (64%)
	Other	5 (6%)
	Do not routinely refer patients	2 (3%)

*Sub-groups may sum to more than 77 (100%) as participants were able to select multiple options

## Discussion

### General considerations

This study describes the development and features of a new PBRN, known as the Chiropractic International Research Collaborative or CIRCuit. It provides an overview of the demographics and practice characteristics of its clinician participants.

### PBRN development

The purpose of CIRCuit is to facilitate the realisation and dissemination of chiropractic-focused, community-engaged research across the world by making it easier for researchers to connect with clinicians. For researchers, CIRCuit can invite clinical collaborators with appropriate demographics and practice characteristics for any particular research project. For clinicians, joining CIRCuit facilitates participating in the undertaking and sometimes development of research projects.

While CIRCuit is international, most other chiropractic PBRNs are nationally based organisations: the Australian Chiropractic Research Network (ACORN) in Australia [24], the Collaborative Research UK Network for Chiropractic (CRUNCh) in the UK [25], one in Canada [5], one in Switzerland [15], and a paediatric-focused one in the USA [26]. In addition, collaborative research groups have been developed to answer specific research questions. Multi-centre cohorts were established to facilitate larger scale data collection in what could be described as PBRNs in Sweden [27], Denmark [28], Norway [29], and the USA [30].

Chiropractic and osteopathic PBRNs have varying missions, often are related to public benefit [5, 15, 24, 25, 31–34]. Other PBRNs take a more profession-focused approach [26, 35].

### Clinician survey

Seventy-seven members (0.7%) of the EBCN responded to the CIRCuit survey designed to recruit clinician participants for the new PBRN.

#### Research engagement

Surveys in Canada in 2008 [22] and 2017 [23] found that about 1% of chiropractors were involved in research. However, 36% of respondents to this study reported research involvement already. This apparent difference noted in our study suggests that respondents are more engaged in research than chiropractors in general, which bodes well for potential sustainability of CIRCuit.

#### Patient visits

CIRCuit respondents reported an average of 55.5 patient visits per week, compared to a study of chiropractors in Ontario, Canada, which found an average of 100 patient visits per week [36]. A study of Australian chiropractors reported an average of 86 patient visits per week [37]. The lower CIRCuit number may reflect international differences in practice styles or could have been affected by the COVID-19 pandemic, as the other studies were conducted before the pandemic.

#### Solo vs. multi-practitioner practice

38% of CIRCuit respondents reported being in solo practice, compared to 50% of Ontario chiropractors [36]. CIRCuit asked about ‘multi-chiropractor’ (reported at 35%) and ‘multi-disciplinary’ practices (reported at 48%), so the true number of CIRCuit chiropractors working with other chiropractors, with or without other practitioners as well, is unknown, but could be as high as 83% (35%+48%). The types of other practitioners included massage therapists, physiotherapists, psychologists, medical doctors, and others. The Ontario study reported that 50% of chiropractors had other chiropractors in the practice, and that 76% had non-chiropractic health care professionals in the practice [36].

#### Academic qualifications

Regarding highest level of qualification attained, some chiropractic courses graduate students at Bachelors level. This is concomitant with medical students in some countries. Many chiropractic courses around the world now graduate students at Masterslevel. We did not seek specificity on type of Masters degrees. Therefore, we do not know if the Masters degrees reported by respondents represented their chiropractic qualification or another, such as a Masters of Public Health or Masters by research.

#### Conditions treated

CIRCuit respondents reported treating back and neck pain ‘often’. Respondents also reported treating extremity musculoskeletal (MSK) conditions often. Similarly, the Ontario study reported back, neck and other MSK conditions as most frequently treated, as did a UK study [38] and the Australian study [37]. 6% of CIRCuit respondents reported often treating non-musculoskeletal disorders; 2% of patient encounters were reportedly to treat non-MSK conditions by Ontario chiropractors [36]. Between 5% and 41% of UK chiropractors ‘strongly agreed’ that non-MSK conditions were ‘effectively treatable by chiropractic methods’, depending on the condition (e.g. infantile colic, asthma, infertility) [38]. The findings herein suggest that conducting practice-based research involving the respondent chiropractors is most feasible exploring MSK rather than non-MSK conditions.

#### Types of therapeutic interventions

95% of CIRCuit respondents reported routinely using high-velocity, low-amplitude manipulation as an intervention. The Ontario study reported ‘manual adjustment’ being used in 72% of patient encounters [36]. Although these two studies report the finding differently, it seems that manipulation of some type is the main intervention used by chiropractors. Most respondents in the CIRCuit, the Ontario study [36] and the Australian study [37] also reported using other therapies, including soft tissue therapy, heat/cold, dry needling/acupuncture and more, as well as offering advice on exercise, lifestyle, nutritional, occupational, and/or pharmaceutical issues. However, such a strong focus on manipulation as a therapeutic intervention raises the question as to whether the evidence supports its use so broadly. Perhaps the evidence for indications and non-indications for joint manipulation should be better developed. Currently, the focus seems to be on contra-indications [39, 40].

#### Profession-specific technique systems

More than half of respondents, 50 (64.9%), reported that they did not use a particular chiropractic technique system (clinical approach). If used, those commonly reported were Activator, Cox flexion-distraction, Thompson, and Gonstead. The most commonly named chiropractic technique in the Ontario study was Activator at 30% of patient encounters, with ‘chiropractic system’ (not further defined) at 20% [36]. The Australian study reported 10% of patient encounters using a ‘chiropractic system’, defined as ‘eg, Applied Kinesiology, Sacro-Occipital Technique, Neuroemotional Technique [37]’.

Although some of these techniques involve a complex, specific system of diagnosis or “subluxation analysis”, it is not known to what extent the full protocols of any technique system are actually employed by practitioners. Anecdotally, for instance, we are aware that an Activator tool, or Gonstead-derived manipulative technique, may be used without employing the other elements of the system.

#### Diagnostic imaging

8% of CIRCuit respondents reported having x-ray in their clinic, and 8% reported having diagnostic ultrasound on site. 3% reported having MRI, and 1% reported having CT. A direct comparison is not possible due to different wording, but the Ontario study reported 12% of practices had ‘diagnostic imaging’ services ‘at the same facility’ [36]. 15% of respondents reported imaging services at the same premises in the Australian study [37].

### Methodological considerations

This study is the first to explore the demographic, practice, and clinical management characteristics of an international cohort of chiropractors interested in participating in research. The lack of external pilot testing with practitioners outside of the committee members is a limitation that could have led to misinterpretation of questions. The study may have been subject to selection bias due to the small sample involved. That is, those who self-selected to participate in the study may be different to non-participants. The sample was small at least partly because participants had to agree to receive invitations to participate in future research projects in order to participate. This could lead to an overrepresentation of chiropractors already involved in research and using evidence-based methods in practice. The electronic data collection approach may have led to under-sampling of clinicians with lower levels of digital literacy. Conceivably, compared to their less literate counterparts, technologically savvy practitioners might be disproportionately more: (a) youthful, or at least young-minded and therefore less tech phobic; (b) recently educated (generally more exposed to evidence-based curricula); and (c) familiar with multi-modal interventions and tools that are accessible online. Recall bias may have also had an effect since we collected information through self-report, rather than directly from clinic records, although recent events are better recalled than distant ones [41]. Since the questionnaire primarily focused on daily or regular activities, this effect is somewhat mitigated. However, some people recall their actions more positively than they actually are [42], which could have led to responses favouring evidence-based practice and research. It was not possible to provide a statistical analysis of the representativeness of the affiliate/practitioner membership within the wider EBCN group or wider chiropractic population more generally but may offer some insight into chiropractors who express an interest in conducting research.

### Implications

PBRNs have the potential to increase meaningful, real-world, community-engaged clinical research. The international nature of the CIRCuit PBRN has the potential to nurture more widespread practice-based research that encompasses a diverse field of social, environmental, and political factors that are often untenable to ascertain in traditional research environments.

In particular, the clinician participants have self-identified as being evidence-based through membership of the EBCN, and demographic results support this. For example, they appear to very frequently use multi-modal care and at-home or supervised exercise interventions, report low to [42]moderate use of diagnostic imaging, and the majority rarely treat non-musculoskeletal conditions. These approaches are all supported by evidence [42–44]. Hence this PBRN may be a useful way for researchers to target practitioners who engage in behaviours that are more consistent with evidence-based practice principles, and to target clinicians more willing to engage in research given that a high proportion (36%) reported being involved in producing research previously. In addition, high proportions of affiliates self-report expertise in chronic pain, headaches, athletic injuries, and dizziness/vertigo, which are all potential areas of interest for researchers.

However, the current cohort of CIRCuit clinician participants may not be representative of the broader EBCN or the profession at large, specific to certain practice and clinical management characteristics (e.g. practice environment, patient visits per week, therapeutic interventions, research engagement). This is unsurprising, given our recruitment targeted a specific sub-population within the profession. Other domains appear to be representative of the EBCN group and the profession as a whole (e.g., conditions treated).

Given that CIRCuit is the first known attempt at creating an international chiropractic PBRN, it is not unexpected that we have encountered challenges in recruiting as deep a pool of clinician participants as we would hope. The current level of 77 clinician participants, spread across the world, may hinder the ability of CIRCuit to assist researchers in each of the world’s regions as effectively as we might wish. In addition, not all regions are represented. Thus, further efforts to invite additional clinician participants as well as representative steering group members from South America, Africa, and Asia would improve diversity and inclusiveness. Focused efforts that utilise multiple pathways or forums to recruit members that extend outside of the EBCN could be considered.

## Conclusions

This paper describes the development and features of a new PBRN for chiropractors. Its mission to facilitate and disseminate research can help increase the research capacity of the profession. The demographic, practice, and clinical management characteristics of the first cohort of clinician participants are described. The international structure is unique among PBRNs and offers the opportunity to help develop innovative research projects.

## Electronic supplementary material

Below is the link to the electronic supplementary material.

Supplementary Material 1

Supplementary Material 2

## Data Availability

Anonymised data are available upon reasonable request.
