# Supplementary material for: Chiropractic international research collaborative (CIRCuit): the development of a new practice-based research network, including the demographics, practice, and clinical management characteristics of clinician participants
Source: Chiropr Man Therap. 2025 Jan 10;33:3. doi: 10.1186/s12998-025-00568-1 (PMC11724568; doi:10.1186/s12998-025-00568-1)
Supplement: Supplementary file 1 — Supplementary Material 1 [file 12998_2025_568_MOESM1_ESM.docx]

**CIRCuit PBRN Participant Questionnaire**

1. **PRELIMINARY INFORMATION**

Questions 1-7 are Acceptance of Terms and Conditions, statement of membership in EBCN, confirmation of current practice status, name, and contact information

1. **PRACTITIONER CHARACTERISTICS**
2. What is your age in years? [enter numerical response]
3. What is your gender identity?

woman / man / non-binary / prefer not to disclose / prefer to self-describe [enter optional response]

1. How many years have you been in clinical practice? Round to the nearest full year. [enter numerical response]
2. Which of the following health professions are you currently a member of?

Chiropractor / Osteopath / Physical therapist / Medical doctor / Nurse / Dietician or Nutritionist / Podiatrist / Occupational therapist / Speech pathologist / Psychiatrist, psychologist, or counsellor / Pharmacist / Emergency medical technician / Other [enter response]

1. What is the highest degree type you currently hold?

Diploma / Bachelor’s Degree / Master’s Degree / Doctor of Chiropractic / PhD / Other [enter response]

1. As a chiropractor, are you involved in any of the following professional activities? Tick as many as apply.

Producing research / Teaching or supervising at an educational institution / Volunteer chiropractic work / Work for a chiropractic organization

1. What language(s) do you routinely consult in? [enter response]

**C. PRACTICE CHARACTERISTICS**

1. What country(s) do you routinely practice in? [enter response]
2. Which of the following best describes your practice location(s)? Tick all that apply.

Urban / Rural / Remote

1. How many separate locations do you routinely practice in? [enter numeral response]
2. Over the past 3 months, what are your average total clinical practice (direct patient contact) hours per week? [enter numerical response]
3. Over the past 3 months, what is your average total number of patient visits per week? [enter numerical response]
4. Over the past three months, what is your average number of NEW patient visits per week?

[enter numerical response]

1. Which of the following best describes the type of practice(s) you work at? Tick all that apply.

Solo Practice / Multi-Chiropractor Practice / Multi-Disciplinary Practice / Hospital-based / Other [enter response]

- 1. If you practice in a multi-disciplinary practice, which types of practitioners routinely work in the practice(s)? Tick all that apply.

Physical therapist / Osteopath / Medical practitioner / Counsellor or Psychologist / Dietician or Nutritionist / Occupational therapist / Speech pathologist / Podiatrist / Fitness professional / Massage therapist / Other [enter response]

1. Which of the following imaging tools or facilities do you have at your practice(s)? Tick all that apply.

X-Ray / Magnetic Resonance Imaging (MRI) / Diagnostic Ultrasound / Computed Tomography (CT) / Other [enter response] / None

1. What type of record-keeping is used at your practice(s)? Tick all that apply.

Primarily paper-based / Primarily electronic / Combination

1. Which of the following best describe the types of payment your practice(s) routinely accept? Tick all that apply.

Private/patient pay / Public health insurance reimbursement / Private health insurance reimbursement / Workers compensation / Personal injury claims / Other [enter response]

**D. PATIENT CARE CHARACTERISTICS**

1. Please indicate the frequency with which you typically treat the following complaints/conditions.

|  | Never/Rarely | Sometimes | Often |
| --- | --- | --- | --- |
| Low back pain  Mid back pain  Neck pain  Radicular symptoms  Headaches  Shoulder pain  Elbow pain  Wrist or hand pain  Hip pain  Knee pain  Calf, ankle, or foot pain  Sports injuries  Postural disorders  Non-musculoskeletal disorders | Ο  Ο  Ο  Ο  Ο  Ο  Ο  Ο  Ο  Ο  Ο  Ο  Ο  Ο | Ο  Ο  Ο  Ο  Ο  Ο  Ο  Ο  Ο  Ο  Ο  Ο  Ο  Ο | Ο  Ο  Ο  Ο  Ο  Ο  Ο  Ο  Ο  Ο  Ο  Ο  Ο  Ο |

1. Please indicate how frequently you treat the following special populations.

|  | Never/Rarely | Sometimes | Often |
| --- | --- | --- | --- |
| Infants (≤1 yr)  Children (2-11 yrs)  Adolescents (12-18 yrs)  Elderly (≥ 60 yrs)  Pregnant females  Athletes  Native/indigenous people  Disabled people | Ο  Ο  Ο  Ο  Ο  Ο  Ο  Ο | Ο  Ο  Ο  Ο  Ο  Ο  Ο  Ο | Ο  Ο  Ο  Ο  Ο  Ο  Ο  Ο |

1. Please indicate categories you feel you have ‘expert’ knowledge/skills to manage. Tick all that apply.

Chronic pain / Headaches / Dizziness and vertigo / Pregnancy and post-partum-related pain / Paediatrics / Athletic injuries/ Other [enter response] / None

1. Please indicate manual treatment techniques you routinely use. Tick all that apply.

High-velocity low-amplitude manipulation / Instrument-assisted joint manipulation / Joint mobilisation / Flexion-distraction / Drop-piece / Pelvic blocking / Soft tissue therapy, trigger point therapy, or massage / Instrument-assisted soft tissue mobilisation / Other [enter response]

1. Please indicate chiropractic technique systems you routinely use. Tick all that apply.

Activator Methods / Sacro-Occipital Technique / Webster Technique / Thompson Technique / Advanced Biostructural Correction Technique / Chiropractic Biophysics / Applied Kinesiology / Gonstead Technique / Cox Flexion-Distraction / Other [enter response] / Do not use a technique system

1. Please indicate adjunct therapies you routinely use. Tick all that apply.

At-home exercise / Supervised exercise / Heat or cold therapy / Rigid taping / Biomechanical taping / Dry needling or acupuncture / Orthotics / TENS / Laser therapy / Therapeutic ultrasound / Other [enter response] / Do not use adjunct therapies

1. Please indicate topics you routinely educate patients about. Tick all that apply.

Physical activity / Sleep hygiene / Stress management / Smoking, drugs, or alcohol / Weight management / Diet or nutrition / Pain science or pain education / workplace modifications/Other [enter response] / Do not use patient education

1. Which practitioners do you routinely refer patients to for any reason? Tick all that apply.

Other chiropractor / Physical therapist / Osteopath / General practitioner / Medical specialist / Dietician or nutritionist / Podiatrist / Occupational therapist / Speech pathologist / Counsellor or psychologist / Fitness professional / Massage therapist / Other [enter response] / Do not routinely refer patients

1. How frequently do you use or refer for diagnostic imaging?

|  | Never/Rarely | Sometimes | Often |
| --- | --- | --- | --- |
| X-Ray  MRI  Ultrasound  CT | Ο  Ο  Ο  Ο | Ο  Ο  Ο  Ο | Ο  Ο  Ο  Ο |
