# Supplementary material for: Chiropractic international research collaborative (CIRCuit): the development of a new practice-based research network, including the demographics, practice, and clinical management characteristics of clinician participants
Source: Chiropr Man Therap. 2025 Jan 10;33:3. doi: 10.1186/s12998-025-00568-1 (PMC11724568; doi:10.1186/s12998-025-00568-1)
Supplement: Supplementary file 2 — Supplementary Material 2 [file 12998_2025_568_MOESM2_ESM.docx]

**Additional file – Responses in free text boxes for the option of ‘other’**

*Practice structure*

Respondents who answered that they worked in a multi-disciplinary practice were asked to specify which other types of practitioners also worked in the practice. Under the category of ‘other’, respondents reported the following types of practitioners: ‘Midwife hypnotherapist’, ‘Kinesiologist, acupuncture’, ‘Cognitive behavioral therapy’, ‘Acupuncturist’, ‘exercise physiologist’, ‘Chinese medical physician’, ‘Registered Acupuncture, Traditional Chinese Medici (sic)’, ‘Acupuncturist’, ‘athletic therapists, TCM’, and ‘Acupuncturist’.

*Self-reported expert knowledge of conditions*

Respondents self-rating of expert level were asked to indicate which conditions they considered themselves to have expert knowledge/skills to manage certain conditions. The responses to ‘other’ comprised the following: ‘Spine Related Disorders/ Post Surgical Spine Rehab’, ‘Spine orthopedics’, ‘Evidence-based care of spine injury’, ‘Concussion’, ‘Extremity problems’, ‘Postural disorders’, and ‘Shoulder pain’.

*Types of manual therapy used*

Respondents were asked about the types of manual therapy they used. The ‘other’ responses to this question included: ‘Rarely use any’, ‘Speeder board (extremities)’, ‘Acupuncture’, ‘Rehabilitation exercise, traction’, ‘Fascial stretch therapy’, ‘Exercise’, ‘Muscle energy techniques’, ‘electric acu’ (sic), and ‘Needling, laser, electro acupuncture, active relea (sic)’.

*Chiropractic technique systems used*

In the free text box after ‘other’, respondents reported the following, regarding chiropractic technique systems they used: ‘Diversidex (sic)/Directional Preference/Neurodynamics’, ‘Diversified’, ‘Motion palpation then any adjustive method’, ‘Diversified’, and ‘Diversified’.

*Adjunct therapies used*

Respondents who answered ‘other’ wrote in the following about therapeutic interventions other than spinal manipulative therapy that they used: ‘shockwave therapy’, ‘dynamic neuromuscular stabilisation’, ‘IASTM’, ‘shockwave’, and ‘radial pulse wave therapy’, ‘Righteye, Senaptec, Interactive Metronome’, ‘PEMF’, ‘percussion’, ‘Vibration, Softwave’, Extra-coporeal (sic) shockwave, PEMF’, ‘Shockwave, interferential current’, and ‘Shock wave, IFT’.

*Patient education*

Additional free text responses on patient education topics included: ‘sleep ergonomics (advice on mattress and pillows)’, ‘MBSR; heat; social life’, ‘posture and proper lifting and sitting’, ‘workload management and recovery’.

*Referral patterns*

Responses specified after the ‘other’ option for referrals to other practitioners included: ‘pain management, neurosurgery’, ‘Acupuncturist’, ‘Chinese medical physician’, ‘Naturopathic doctor’, and ‘Bach flower remedies’.
